# The Role of Paternal Parenting and Co-parenting Quality in Children’s Academic Self-Efficacy

**DOI:** 10.3389/fpsyg.2022.772023

**Published:** 2022-03-21

**Authors:** Demet Kara, Nebi Sümer

**Affiliations:** ^1^Department of Psychology, Middle East Technical University, Ankara, Turkey; ^2^Faculty of Arts and Social Sciences, Sabancı University, Istanbul, Turkey

**Keywords:** fathers, paternal and maternal parenting behaviors, parenting consistency, coparenting, academic self-efficacy

## Abstract

This study explored the unique effect of fathers’ parenting behaviors and the quality of co-parenting described as the degree of consistency between paternal and maternal parenting behaviors on children’s academic self-efficacy. The power of both pancultural parenting behaviors (i.e., emotional warmth and rejection) and specific parenting controlling behaviors that are relatively common in Turkish culture (i.e., intrusion and guilt induction) in predicting academic self-efficacy was tested. A total of 1,931 children completed measures of parenting behaviors and academic self-efficacy in math and literature courses in their school. Overall, girls reported higher levels of literature self-efficacy, whereas boys reported higher levels of math self-efficacy. Compared to boys, girls perceived higher levels of positive parenting behaviors from both their fathers and mothers. The results of the regression analyses showed that, whereas father warmth had stronger effects on boys’ math self-efficacy, mother warmth had stronger effects on girls’ literature self-efficacy. Examination of the effects of co-parenting quality demonstrated that children with positively consistent parents (i.e., both parents having high positive and low negative parenting behaviors) reported the highest level of academic self-efficacy, whereas those having negatively consistent parents had the lowest level of academic self-efficacy. Analyses on inconsistent co-parenting, however, yielded compensatory effects, which were similar to positively consistent parents, and deterioration effects, which were similar to negatively consistent parents depending on the gender of parent and child, domain of parenting behavior, and academic efficacy. This study contributed to the current literature by showing the unique role of fathers over and beyond mothers, and confirmed the importance of positive parenting and parenting consistency in promoting children’s academic efficacy. Cultural and practical implications of the findings were discussed.

## Introduction

Because of rapid social changes, especially in women’s status, fathers’ traditional gendered role as breadwinners and conveyers of moral values have been globally transformed into more egalitarian roles (Lamb, 2000). Consequently, fathers have been more active in caregiving and co-parenting in recent decades (Jones and Mosher, 2013; National Survey of Family Growth, 2017). Today’s fathers are more emotionally available, and their role in child development has evolved beyond only providing material or instrumental support (Cabrera et al., 2000; Lamb, 2000). These changes have been mostly achieved with recent attempts calling attention to fathers’ role and the importance of their engagement in raising children. For instance, past studies have documented that children show substantial progress when fathers are actively involved in their children’s academic-related topics. In a recent meta-analysis, Kim and Hill (2015) found that both fathers’ and mothers’ educational engagement contributed to student achievement. Similarly, Jeynes (2015) showed that father involvement predicted academic and psychological outcomes from kindergarten to undergraduate years. Past studies, however, have not examined the unique effect of fathers, compared to mothers, as well as effects of parental consistency as an indication of co-parenting quality on school children’s academic self-efficacy. Thus, in this study, with an emphasis on paternal parenting, we explored the effect of two universal (relatively pancultural) parenting behaviors, namely, emotional warmth and rejection, and two parental psychological controlling behaviors, intrusion and guilt induction, which are relatively common in the Turkish context on children’s academic self-efficacy. We expected that girls’ and boys’ perceptions of positive, negative, and (in)consistent paternal and maternal behaviors would have distinct effects on their academic self-efficacy.

Specifically, the first aim of this study was to extend fathering literature by investigating the unique contribution of paternal parenting behaviors in the effect of maternal behaviors. The second goal was to examine if and how children’s level of academic self-efficacy in math and literature courses changes as a function of co-parenting quality considering (in)consistency between paternal and maternal behaviors. We tested our hypothesis separately for girls and boys.

### Unique Role of Fathers

The visibility of fathers in child development is growing. Fathers are publicly informed about positive impacts of their presence on child functioning (Cabrera and Peters, 2000). Community programs and policies take serious actions to encourage fathers to become more involved in their children’s lives (Tully et al., 2017). A past study has extensively documented fathers’ critical role in children’s cognitive, social, and educational developments across cultures and developmental periods (see Jeynes, 2015; Rollè et al., 2019 for reviews). For instance, in the United States, early adolescents having involved fathers have lower levels of internalizing and externalizing problems compared to those with uninvolved fathers (Day and Padilla-Walker, 2009). Similarly, among Mexican immigrant families, although mothers spend more time with childcaring, fathers’ time spent in academic care increases children’s academic performance (Hossain and Shipman, 2009). Overall, father and mother involvement were equally associated with students’ academic achievement (Kim and Hill, 2015). Past studies conducted in the Turkish context also supported the positive impact of father involvement. For instance, the quality of father-daughter relationship was a strong predictor of adolescents’ well-being (Sağkal et al., 2018). Moreover, both mothers’ and fathers’ parenting behaviors separately predicted primary school students’ academic performance, although mother effect was stronger (Erdoğdu, 2007).

A previous study has suggested a number of qualitative and quantitative differences between fathers’ and mothers’ roles in various child outcomes across developmental stages. For instance, Chen et al. (2000) found that mothers and fathers contributed to different developmental outcomes in the Chinese context, whereas maternal warmth was related to child emotional adjustment, paternal warmth was related to social and academic achievements. Verhoeven et al. (2012) found that mother and father parenting dimensions had unique effects on child anxiety across different periods; maternal over-control was more predictive of anxiety in early years, whereas paternal over-control was more predictive during adolescence. In another study, Lv et al. (2018) examined the effect of parental involvement on children’s multidimensional (i.e., academic, emotional, and social) self-efficacy profiles. They found that the effect of fathers’ and mothers’ educational aspirations varies across different self-efficacy profiles. Fathers’ educational aspirations predicted children’s high self-efficacy profiles, while mothers’ educational aspirations prevented children to be in the low level of self-efficacy profile. These observed differences seem to stem from different functions of maternal and paternal parenting goals and strategies. Mothers mainly focus on providing emotional support and nurturing, while fathers mostly provide guidance to their children about future behaviors (Jeynes, 2016). In a meta-analytic study, Jeynes (2015) showed that although both fathers and mothers affect child development through different pathways, fathers’ unique role was held for both younger and older children as well as for girls and boys, especially in academic achievement.

Documented differences between mother and father effects may depend on the way children relate to their parents. For instance, Turkish adolescents perceive different levels of affection, control, autonomy, and discipline from their parents. Children perceive their mothers as more affectionate than their fathers, while they perceive their fathers as more controlling, disciplining, and autonomy-granting than their mothers (Sunar, 2009). One of Turkey’s largest foundations supporting positive parenting, AÇEV [Mother Child Education Foundation (Anne C̨ocuk Eğitim Vakfı, 2017)] published a comprehensive report titled “Involved fatherhood and its determinants in Turkey” in 2017. This report shows that traditional fatherhood defined with characteristics of patriarchal authoritarian parenting is still common in Turkey. However, there also exists an emerging *new traditional fatherhood*. Fathers of this type are similar to traditional fathers in their attitudes toward masculinity but different from them in showing more affection to their daughters in their relationships. These two types of traditional fatherhood are the most prevalent types in Turkey. Moreover, as an optimal type, involved fathering is characterized by care, control, and affection and is seen in metropolitan cities among egalitarian families.

Collectivistic values of Turkish culture still characterize fatherhood roles in Turkey as being less emotionally but more instrumentally and financially involved (Ataca, 2009). These characterizations seem to affect children’s perception of maternal and paternal parenting (Sümer and Kağıtc̨ıbas̨ı, 2010). Therefore, in this study, we mainly aimed to investigate how perceived paternal parenting behaviors affect children’s academic self-efficacy over perceived maternal parenting behaviors. To better understand fathers’ unique parenting role in child academic self-efficacy, we systematically compare it with mothers’ effect. Fathers’ involvement and parenting behaviors are also critical for harmony (consistency) between parents as well as coparenting (Jia and Schoppe-Sullivan, 2011; Fagan and Cabrera, 2012). Thus, we specifically examine the effect of (in)consistency between paternal and maternal parenting on child academic self-efficacy.

### Co-parenting

While some couples with children display a full agreement or harmony in child rearing, others may diverge and adopt different styles. The similarities or differences observed between mothers and fathers may stem from certain factors, such as the level of agreement between parents on child-rearing strategies (Feinberg, 2003), traditional gender roles of parents in a given culture (Craig and Mullan, 2011), and marital discord (Margolin et al., 2001) though not limited to these factors only. Co-parenting indicates parents’ consistent behaviors, overlapping strategies, and shared responsibilities in child rearing (Feinberg, 2003). It is a central process for child adjustment (Margolin et al., 2001). Thus, parents need to display mutual support and coordinate their behaviors for optimal child outcomes. A past study has shown that besides mothers’ and fathers’ individual parenting styles (Fan and Chen, 2020), co-parenting is also related to other aspects of family dynamics such as marital conflict (Margolin et al., 2001; Schoppe-Sullivan et al., 2004) and parental divorce (Maccoby et al., 1990; Becher et al., 2019).

Co-parenting is commonly characterized by cooperation, support, sharing of responsibilities, and agreement between parents on child rearing issues (Teubert and Pinquart, 2010). In this study, we extend the definition of co-parenting by adding consistency in parental behaviors. Specifically, we define three types of co-parenting consistency based on four categorizations of paternal and maternal parenting considering its potential impact on child outcomes. The first type is *positive coparenting* in which both mothers and fathers show the most adaptive level of coordination by simultaneously adopting ideal parenting behaviors. For instance, both parents show high levels of emotional warmth or low levels of rejection in this type. As the optimal type, positive co-parenting is assumed to improve child functioning and promote favorable child outcomes. The second one is *negative coparenting* in which both parents simultaneously adopt dysfunctional parenting behaviors or understate adaptive parenting practices. For instance, both parents show low emotional warmth and high rejection behaviors. The final type is *inconsistent coparenting* in which parents adopt discrepant levels of the same parenting behaviors as one parent is showing high and the other one is showing low levels of the same parenting behavior. These parents may not be motivated to act synchronized or, conversely, overdue the role of the other parental figure within the family.

The quality of these co-parenting behaviors may lead to various outcomes. We can speculate that while positive co-parenting is the most adaptive, negative co-parenting undoubtedly is the most dysfunctional one. Inconsistent co-parenting, however, may fall in between, and its effect may vary depending on which parent, mother or father, has the higher or lower level of the given parenting behavior in a culture-specific context. That is, having one parent who fits well with the developmental needs of children can compensate the other parent’s incongruent parenting behavior and protect children from potential negative outcomes. We define the potential effect of this type as the *inconsistency compensation* effect. Depending on the child’s gender and specific parenting behavior, we can observe either *mother compensatory* or *father compensatory* effect. Nevertheless, having one parent with less ideal behaviors within a parenting dyad is still a risk factor. Such inconsistent parenting behaviors might lead to identical outcomes with negative coparenting. That is, the presence of one parent’s negative behavior could be enough to produce negative outcomes. We call this type of effect the *inconsistency deterioration* effect. Again, depending on the child’s gender and specific parenting behavior, we can observe either *mother deterioration* or *father deterioration* effect. Effects of inconsistent co-parenting can be sensitive to cultural contexts. For instance, in a traditionally gendered culture in which mothers play a nurturing role and fathers play a strict disciplining (controlling) role, low maternal warmth or high maternal rejection may lead to more harmful outcomes than low paternal warmth or high paternal rejection.

Indeed, convergent evidence supports these claims. For instance, 2-year-old children who have one supportive parent were more advantaged in their cognitive development than those who have none (Ryan et al., 2006). Co-parenting conflicts negatively predicted preschool children’s math and literacy scores (Cabrera et al., 2012), increased the development of adolescents’ risky behaviors (Baril et al., 2007), or predicted higher levels of adolescents’ antisocial behaviors (Feinberg et al., 2007). Moreover, a previous study has shown a relationship between parental (dis)agreement and (in)consistencies, and children’s psychological controlling (Block et al., 1981), ego resiliency (Lamb et al., 1989), moral adjustment, personality development (Vaughn et al., 1988), and psychological disorders (Dwairy, 2008). In their meta-analytic study, Teubert and Pinquart (2010) thoroughly examined the role of co-parenting, particularly coordination and agreement, in child and adolescent internalizing and externalizing problems as well as social functioning. Their findings revealed that co-parenting had stronger effects on longitudinal change in child adjustment levels. Considering co-parenting influences a large number of developmental outcomes; this study tested its role in one of those, namely, academic self-efficacy.

### The Relationship Between Parenting and Academic Self-Efficacy

Academic self-efficacy or academic self-concept is the individuals’ knowledge and perceptions about their performance in academic situations (Ferla et al., 2009). We used academic self-efficacy and academic self-concept interchangeably, although others argue differences between these concepts (see Marsh, 1990). Individuals’ beliefs and perceptions vary across academic subjects in interaction with gender, such that boys are generally more confident in mathematics, science, or areas related to technology, and girls have either higher levels of self-efficacy in language and literacy than boys or have similar levels of self-efficacy even though girls’ actual performance is better (Pajares, 2003; Pajares et al., 2007). As a motivational basis of academic success, academic self-efficacy refers to students’ attitudes and mastery beliefs in academic domains and is a strong predictor of subsequent academic achievement (Marsh and Martin, 2011; Marsh and Seaton, 2013). There exists a bidirectional relationship between academic self-concept and achievement (see Marsh and Martin, 2011, for a discussion), suggesting that academic self-efficacy is both dependent on previous performance (Ferla et al., 2009) and helps in increasing the current level of achievement (Marsh and Martin, 2011). The way parents exert power and control on their children or provide feedback and appreciation to them is also critical for academic self-efficacy and school success. A previous study has demonstrated that school-specific parenting behaviors (Catsambis, 2001), parenting control (Masud et al., 2015; Pinquart, 2016), and parental involvement in children’s academic engagement (Cheung and Pomerantz, 2011) have a robust effect on children’s academic success, and that academic self-efficacy mediates the effect of parenting styles on academic performance (Llorca et al., 2017).

Still, the vast majority of previous studies obtained single parent reports only; thus, there is scarcity in studies examining the separate, combined, and additive effects of fathers and mothers in terms of co-parenting consistency. In a recent study, Suizzo et al. (2017) found a unique effect of fathers’ warmth on adolescents’ academic development. Indeed, in their study, positive paternal behaviors such as father warmth influenced adolescents’ academic performance by increasing positive beliefs such as optimism and academic self-efficacy as well as their level of determination (Suizzo et al., 2017). We propose that, over and beyond mothers’ effect, fathers could influence children’s academic self-efficacy. In addition to emotional warmth, we included three more parenting behaviors, which are rejection, intrusion, and guilt induction. Specifically, we examined if the predictive power of paternal behaviors on girls’ and boys’ academic self-efficacy varies in specific dimensions of parenting behaviors. For instance, Pinquart (2016) found that school-specific parenting behaviors had stronger effects than general parenting styles. As would be expected, authoritative parenting was more effective in increasing children’s academic performance than other parenting styles (Masud et al., 2015). In this study, we included both relatively global (or culture free) parenting behaviors, namely, parental emotional warmth and rejection, and culture-specific (i.e., relatively common in Turkish culture) parental psychological control behaviors, namely, guilt induction and intrusion, to systematically investigate the effects of critical parenting behaviors on the domains of academic self-efficacy. Previous studies have also provided some evidence for the interaction between parent and child gender by comparing same-sex parent–child dyads with mixed-dyads (Pinquart, 2016). Although this was beyond our purposes, we performed separate analyses for girls and boys considering that their academic self-efficacy differs across academic domains.

### Universal vs. Culture-Common Parenting Behaviors

Parents adopt different child-rearing strategies across cultures (Bornstein, 2012). Some strategies manifest similar positive or negative effects on children regardless of cultural setting, although others’ effects are bound to specific cultural contexts. These culturally bound parenting behaviors are considered less desirable in universal terms, although they may be compatible with cultural values and parents’ socialization goals in a given cultural context. Thus, certain parenting behaviors become relatively more normative and less harmful in specific cultures. Parental psychological control is a typical example of culture-specific effects. In collectivistic cultures like that in China, parents frequently rely on components of psychological control such as love withdrawal, shaming, or guilt induction as parenting methods (Olsen et al., 2002). Although parental psychological control is generally considered a harmful practice in Western cultures, parents in the East may use the means of psychological control to socialize with their children in line with cultural values (Scharf and Goldner, 2018). For instance, whereas Chinese mothers’ academic involvement was accompanied with higher levels of psychological control, American parents’ academic involvement was accompanied with higher levels of autonomy support, and still, parental involvement predicted children’s increased level of academic engagement and achievement in both cultures (Cheung and Pomerantz, 2011). That is, intrusive parenting strategies are relatively common and not perceived as harmful in mainly collectivistic cultures.

Parents in Turkish culture with their closely knit family structure and collectivistic values have traditional child socialization goals and parenting practices, which are mainly characterized by parental control (Kağıtc̨ıbas̨ı, 2007). For instance, it is a relatively common practice to adopt certain psychological controlling behaviors such as guilt induction, using guilt as a means of pressuring children to comply with parental demands (Barber and Harmon, 2002), or intrusion. However, these practices are not necessarily perceived as negative and may even be perceived as a sign of involvement and care, as well as a way of transmitting expectations. Similarly, Rudy and Halgunseth (2005) showed that guilt induction is common in collectivist cultures and is not related to maladaptive parental cognitions. Sümer and Kağıtc̨ıbas̨ı (2010) identified three dimensions of psychological control, namely guilt induction, love withdrawal, and overprotection, as culturally relevant or culture-common behaviors in Turkey and examined their effects with parental warmth and rejection on attachment to parents during middle childhood. Results showed that although parental warmth and rejection, representing universal patterns, strongly predicted attachment to parents, the three subdimensions of psychological control either had no effects or had weak negative effects on attachment to parents. These findings suggest that certain aspects of parental psychological control such as mild intrusion might be perceived as normative in Turkish culture. However, we need to be cautious in these arguments, since effects of culture-common parenting behaviors vary, and contradictory findings exist (e.g., Bean et al., 2003; Kindap et al., 2008).

### This Study

The aim of this study was twofold. First, we examined the unique role of fathers’ parenting behaviors in girls’ and boys’ academic self-efficacy in math and literature courses. We specifically focused on common (i.e., parental intrusion and guilt induction) and universal (i.e., emotional warmth and rejection) parenting behaviors. Overall, we expected that parental warmth positively predicts but rejection, intrusion, and guilt induction negatively predict literature and math self-efficacy. The effect of paternal parenting behaviors remains significant over and beyond the effect of matched maternal parenting behaviors. Second, we investigated the effect of co-parenting quality on girls’ and boys’ levels of academic self-efficacy. Academic self-efficacy was expected to differ according to the quality of perceived co-parenting behaviors. We specifically proposed that positive co-parenting behaviors are related to highest levels of academic self-efficacy, and that negative co-parenting behaviors are related to lowest levels of academic self-efficacy in both literature and math courses. Effects of inconsistent co-parenting behaviors were expected to fall in between these two ends. On the one hand, this effect would be similar to positive co-parenting if an inconsistency compensatory effect exists. Considering gender-based parenting roles (i.e., nurturing mothers and controlling fathers) in Turkish culture, we expected that compensatory effects would particularly be seen for culture-common parenting dimensions. On the other hand, it would be similar to negative co-parenting if an inconsistency deterioration effect exists. We expected that deterioration effects would be more likely for universal parenting dimensions given that they refer to the value of the child in the family (i.e., parental warmth has positive and rejection has negative effects regardless of cultural variation and parent’s gender). Finally, although we did not have specific hypotheses or test the interaction effect between parent’s or child’s gender and outcome variables, we still expected to observe a joint effect. That is, there would be father compensatory or deterioration effects on boys’ math self-efficacy and mother compensatory or deterioration effects on girls’ literature self-efficacy.

## Materials and Methods

### Participants

This study was part of a larger community-based study conducted by Sümer et al. (2009). Data were collected from 4th^–^ and 5th-grade children across 16 schools from different cities in Turkey (i.e., Ankara, Samsun, Mersin, and Manisa). All the 4th^–^ and 5th-grade children from selected schools were targeted as sample. Children whose parents agreed on their participation and signed the consent form participated to the study. Overall, there were 1,931 children in the final sample (*M*_age_ = 10.37 years, *SD* = 0.88). Gender and age distribution of the participants (*N*_girls_ = 978, *M*_age_ = 10.36 years, *SD* = 0.9; *N*_boys_ = 953, *M*_age_ = 10.38 years, *SD* = 0.87) were almost equal. The children rated parenting behaviors of their mothers (*M*_age_ = 36.48, *SD* = 5.11) and their fathers (*M*_age_ = 40.83, *SD* = 5.79). Majority of the mothers were primary school (29.7%) or high school graduates (30.8%), followed by university (19.9%) and middle school (13.6%) levels. A small percentage of mothers (2.6%) did not have any formal education. Regarding fathers, majority of them were high school (30.2%) or university graduates (30.5%), followed by primary (18%) and middle school (15.3%) levels. A small percentage of the fathers (0.8%) did not have any formal education. Besides, 3.4% of mother education data and 5.2% of father education data were missing.

### Measures

Parenting behaviors were measured through a collection of parenting scales used in Sümer et al. (2009) study. The scales and items explained below were adapted from different measures or composed by the researchers (Barber, 1996; Arrindell et al., 1999; Olsen et al., 2002; Sümer et al., 2009). These measures aimed to assess children’s perceptions about their mothers’ and fathers’ parenting behaviors on the emotional warmth, rejection, intrusion, and guilt induction dimensions. Children completed the same measures for their mothers and fathers separately on a 4-point Likert scale (1 = no, 2 = sometimes, 3 = most of the time, and 4 = always).

The emotional warmth and rejection dimensions represent universal parenting behaviors, whereas intrusion and guilt induction represent critical dimensions of parental psychological control that are not uncommon in the Turkish cultural context. Therefore, these two dimensions are briefly labeled as culture-common parenting behaviors (see Sümer and Kağıtc̨ıbas̨ı, 2010). This study adopted a predefined factor structure performed by Sümer et al. (2009) supporting the psychometric quality of the measures.

The universal dimension of parenting, emotional warmth, and rejection subscales was measured using Arrindell et al. (1999) corresponding subscales in the EMBU. The warmth subscale (8 items, α = 0.8 and α = 0.81 for mothers and fathers, respectively) measures positive parenting behaviors such as unconditional love, special care, or being interested in children’s needs and desires (e.g., *Does your mother/father try to comfort you when something bad happened?*). The rejection subscale (11 items, α = 0.84 and α = 0.88, for mothers and fathers, respectively) measures parents’ insensitivity to their children’s needs and desires as well as the level of perceived punishment or conflict (e.g., *Does your mother/father get tough on you?*). Subscales for intrusion and guilt induction were developed by Sümer et al. (2009) considering related parenting behaviors that are common in Turkish culture. The eight-item intrusion subscales (α = 0.69 and α = 0.71 for mothers and fathers, respectively) assess how much parents interfere with their children’s autonomy with intrusive behaviors (e.g., *Does your mother/father move your stuff in your room without asking?*). The guilt induction subscale (5 items, α = 0.45 and α = 0.46 for mothers and fathers, respectively) measures parents’ intention to make their children feel guilty about their undesirable behaviors (e.g., *Do you feel that you have disappointed your mother/father?*). Reliability coefficients for the guilt induction subscale were relatively low, since they consist of a few items assessing different aspects of guilt-inducing parenting behaviors.

Academic self-efficacy was measured *via* Turkish translation (Özdemir, 2002) of the Academic Self-Description Questionnaire (ASDQ) developed by Marsh (1990). This is a self-rating instrument composed of two dimensions, literature self-efficacy and math self-efficacy, and 6 items for each dimension (e.g., *When I compare myself with my peers, I am good at Literature/Math.*). Children rated themselves on these items with a 4-point scale (1 = completely false, 2 = false, 3 = true, 4 = completely true). Internal consistency coefficients for literature (α = 0.83 and α = 0.82 for girls and boys, respectively) and math subscale (α = 0.85 and α = 0.84, for girls and boys, respectively) were high in this study.

### Procedure

A set of questionnaires was given to children after obtaining a consent form from their parents. They responded to the parenting behavior scale separately for their mothers and fathers. They also evaluated their academic self-efficacy in literature (Turkish) and math courses. All procedures and materials were approved by Middle East Technical University, Human Research Ethics Committee.

## Results

### Statistical Method

All statistical analyses were conducted using IBM SPSS Statistics 26 (IBM Corp, 2019; Armonk, NY, United States). We first performed descriptive statistics with *t*-tests and correlation analysis. We then performed a hierarchical regression analysis to test the predictive power of paternal parenting behaviors over and beyond the effect of maternal parenting behaviors. Lastly, we performed a series of ANOVA to test the role of co-parenting quality in children’s literature self-efficacy (LSE) and math self-efficacy (MSE).

### Descriptive Statistics

#### Gender Differences in Study Variables

We first examined potential gender differences on the study variables *via* a series of one-way ANOVAs. As seen in Table 1, the girls reported higher levels of LSE than the boys [*F*(1,1,911) = 74.22, *p* < 0.001], whereas the boys reported higher levels of MSE than the girls [*F*(1,1,911) = 15.18, *p* < 0.001]. In addition, the girls perceived higher levels of positive parenting behaviors from both fathers and mothers. That is, the girls reported higher levels of paternal warmth [*F*(1,1,906) = 4.47, *p* < 0.05] and maternal warmth from their parents than the boys [*F*(1,1,924) = 16.6, *p* < 0.001]. However, this pattern was reversed for negative parenting dimensions. That is, the boys reported higher levels of paternal rejection [*F*(1,1,905) = 29.92, *p* < 0.001] and maternal rejection than the girls [*F*(1,1,924) = 13.32, *p* < 0.001]. Also, the boys reported higher levels of paternal intrusion [*F*(1,1,905) = 47.79, *p* < 0.001] and maternal intrusion than the girls [*F*(1,1,923) = 34.75, *p* < 0.001]. Perceived paternal guilt induction was higher for the boys than for the girls [*F*(1,1,905) = 15.16, *p* < 0.001]; however, perceived maternal guilt induction was marginally different [*F*(1,1,924) = 3.83, *p* = 0.051].

**TABLE 1:** Intercorrelations between the study variables and means and standard deviations (SD).

	1	2	3	4	5	6	7	8	9	10
(1) LSE	1	0.46**	−0.18**	−0.15**	0.28**	0.24**	−0.10**	−0.10**	0.02	0.03
(2) MSE	0.39**	1	−0.25**	−0.21**	0.26**	0.29**	−0.22**	−0.15**	−0.00	0.06
(3) Mother Rejection	−0.18**	−0.17**	1	0.49**	−0.39**	−0.30**	0.62**	0.37**	0.18**	0.16**
(4) Father rejection	−0.17**	−0.17**	0.53**	1	−0.22**	−0.44**	0.36**	0.67**	0.14**	0.11**
(5) Mother warmth	0.34**	0.20**	−0.40**	−0.23**	1	0.60**	−0.25**	−0.11**	0.26**	0.18**
(6) Father warmth	0.37**	0.25**	−0.30**	−0.42**	0.64**	1	−0.23**	−0.28**	0.11**	0.26**
(7) Mother intrusion	−0.09**	−0.16**	0.62**	0.42**	−0.22**	−0.18**	1	0.50**	0.21**	0.17**
(8) Father intrusion	−0.13**	−0.14**	0.41**	0.68**	−0.13**	−0.27**	0.57**	1	0.18**	0.17**
(9) Mother guilt	0.08*	0.02	0.14**	0.13**	0.31**	0.18**	0.24**	0.18**	1	0.64**
(10) Father guilt	0.11**	0.03	0.15**	0.21**	0.23**	0.25**	0.24**	0.27**	0.62**	1
Means (*SD*) for girls	3.40 (0.49)	3.09 (0.57)	1.18 (0.29)	1.16 (0.32)	3.48 (0.52)	3.35 (0.60)	1.41 (0.38)	1.29 (0.36)	2.31 (0.59)	2.23 (0.58)
Means (*SD*) for boys	3.20 (0.53)	3.19 (0.55)	1.24 (0.36)	1.25 (0.38)	3.38 (0.55)	3.29 (0.60)	1.52 (0.46)	1.42 (0.43)	2.36 (0.59)	2.33 (0.60)

*Upper diagonal represents correlation coefficients for girls and lower diagonal represents correlation coefficients for boys.*

*p < 0.05, **p < 0.01.

#### Bivariate Correlations

Correlations among study variables are presented in Table 1. All perceived parenting variables, except guilt induction, were significantly associated with LSE and MSE for both girls and boys. Both universal parenting dimensions (warmth and rejection) were strongly correlated with girls’ and boys’ academic self-concept (LSE and MSE). That is, LSE and MSE were positively correlated to warmth and negatively correlated to rejection. However, culture-common parenting behaviors (guilt induction and intrusion) were weakly correlated with the same outcome variables. Both girls’ and boys’ LSE and MSE were negatively correlated with intrusion, whereas the boys’ LSE was positively correlated with guilt induction.

### Testing the Predictive Power of Paternal Parenting Variables

We performed four sets of hierarchical regression analyses to test the predictive power of paternal parenting on girls’ and boys’ literature and math self-efficacy over and above maternal parenting variables. As presented in Table 2, we first tested the effect of maternal and paternal parenting behaviors on girls’ LSE levels. The models were significant in the first step [*F*(4,952) = 22.2, *p* < 0.001, *R*^2^ = 0.085] and in the second step [*F*(8,948) = 22.28, *p* < 0.001, *R*^2^ = 0.094]. Results revealed that mother warmth positively and rejection negatively predicted girls’ LSE in the first step. Mother warmth remained significant in the second step [*B* = 0.21, *t*(948) = 4.88, *p* < 0.001]. There were no other significant effects.

**TABLE 2:** Maternal and paternal parenting behaviors predicting girls’ and boys’ literature and math self-efficacy.

	Literature self-efficacy				Math self-efficacy			
		
		
	Girls		Boys		Girls		Boys	
				
	Beta (*SE*)	*B* (Bootstrapped 95% CI)	Beta (*SE*)	*B* (Bootstrapped 95% CI)	Beta (*SE*)	*B* (Bootstrapped 95% CI)	Beta (*SE*)	*B* (Bootstrapped 95% CI)
**Step 1**								
Mother warmth	0.24 (0.03)	0.26** (0.19 – 0.32)	0.32 (0.04)	0.33** (0.26 – 0.40)	0.21 (0.04)	0.19** (12 – 0.28)	0.17 (0.04)	0.17** (0.09 – 0.24)
Mother rejection	−0.15 (0.07)	−0.09* (−0.19 – [−0.00])	−0.06 (0.06)	−0.04 (−0.13 – 0.05)	−0.20 (0.08)	−0.10* (−0.22 – [−0.03])	−0.07 (0.07)	−0.05 (−0.13 – 0.05)
Mother intrusion	0.04 (0.05)	0.03 (−0.05 – 0.11)	0.00 (0.05)	0.00 (−0.08 – 0.09)	−0.15 (0.06)	−0.10* (−0.21 – [−0.02])	−0.11 (0.05)	−0.09* (−0.17 – [−0.01])
Mother guilt induction	−0.03 (0.03)	−0.04 (−0.10 – 0.02)	−0.01 (0.03)	−0.01 (−0.08 – 0.06)	−0.01 (0.03)	−0.01 (−0.09 – 0.06)	0.00 (0.03)	0.00 (−0.07 – 0.07)
**Step 2**								
Mother warmth	0.20 (0.04)	0.21** (0.12 – 0.30)	0.18 (0.04)	0.19** (0.19 – 0.28)	0.11 (0.05)	0.10* (0.01 – 0.20)	0.06 (0.05)	0.06 (−0.03 – 0.15)
Mother rejection	−0.12 (0.08)	−0.07 (−0.18 – 0.02)	−0.05 (0.07)	−0.04 (−0.13 – 0.05)	−0.16 (0.09)	−0.08^†^ (−0.19 – 0.01)	−0.05 (0.07)	−0.03 (−0.12 – 0.06)
Mother intrusion	0.07 (0.06)	0.05 (−0.03 – 0.14)	0.03 (0.05)	0.03 (−0.06 – 0.12)	−0.15 (0.06)	−0.10* (−0.21 – [−0.00])	−0.09 (0.06)	−0.08^†^ (−0.16 – 0.02)
Mother guilt induction	−0.03 (0.04)	−0.03 (−0.11 – 0.05)	−0.03 (0.04)	−0.04 (−0.13 – 0.05)	−0.05 (0.04)	−0.05 (−0.14 – 0.05)	0.00 (0.04)	0.00 (−0.09 – 0.09)
Father warmth	−0.07 (0.04)	0.08^†^ (−0.00 – 0.17)	0.19 (0.04)	0.22** (0.13 – 0.31)	0.14 (0.04)	0.15** (0.06 – 0.18)	0.16 (0.04)	0.18** (0.08 – 0.26)
Father rejection	−0.04 (0.07)	−0.03 (−0.13 – 0.07)	0.00 (0.07)	0.00 (−0.09 – 0.09)	−0.13 (0.08)	−0.07 (−0.20 – 0.03)	−0.03 (0.07)	−0.02 (−0.10 – 0.07)
Father intrusion	−0.04 (0.06)	−0.03 (−0.14 – 0.07)	−0.06 (0.06)	−0.05 (−0.15 – 0.05)	0.05 (0.07)	0.03 (−0.08 – 0.14)	−0.02 (0.06)	−0.02 (−0.11 – 0.07)
Father guilt induction	0.01 (0.04)	0.01 (−0.08 – 0.09)	0.04 (0.04)	0.05 (−0.04 – 0.13)	0.07 (0.04)	0.07 (−0.03 – 0.15)	0.00 (0.04)	0.00 (−0.08 – 0.09)

^†^p < 0.10, *p < 0.05, **p < 0.01.

Regression analysis on boys’ level of LSE was significant in the first step [*F*(4,928) = 32.04, *p* < 0.001, *R*^2^ = 0.121] and in the second step [*F*(8,924) = 21.77, *p* < 0.001, *R*^2^ = 0.159]. Mother warmth significantly and positively predicted the boys’ LSE both in the first step and in the second step [*B* = 0.19, *t*(924) = 4.33, *p* < 0.001]. Father warmth also significantly and positively predicted the boys’ LSE levels [*B* = 0.22, *t*(924) = 4.97, *p* < 0.001]. These results suggested a significant additive effect of father warmth over and beyond the effect of mother warmth.

We tested the role of perceived maternal and paternal parenting variables in girls’ MSE levels in the third model analysis. The models were significant in the first step [*F*(4,952) = 25.68, *p* < 0.001, *R*^2^ = 0.097] and in the second step [*F*(8,948) = 16.83, *p* < 0.001, *R*^2^ = 0.124]. Results revealed significant positive effects of mother warmth and negative effects of mother rejection and mother intrusion in the first step. The mother warmth [*B* = 0.1, *t*(948) = 2.32, *p* < 0.05] and mother intrusion [*B* = −0.1, *t*(948) = −2.31, *p* < 0.05] variables remained statistically significant in the second step. Also, father warmth significantly and positively predicted girls’ MSE levels [*B* = 0.15, *t*(948) = 3.37, *p* < 0.001].

The final regression analysis on boys’ MSE levels yielded significant models in the first step [*F*(4,928) = 14.08, *p* < 0.001, *R*^2^ = 0.057] and in the second step [*F*(8,924) = 9.92, *p* < 0.001, *R*^2^ = 0.079]. Mother warmth had a positive significant effect and mother intrusion had a negative significant effect on boys’ MSE levels in the first step. However, these effects were not significant in the second step. Father warmth significantly and positively predicted boys’ MSE levels [*B* = 0.18, *t*(924) = 3.82, *p* < 0.001]. There were no other significant effects. Paternal emotional warmth was the most critical predictor of boys’ math efficacy.

Overall, the findings suggested that all the four models were significant both in the first and second steps, indicating the additive effects of paternal parenting behaviors above and over maternal parenting to be in line with our expectations. However, only perceived father warmth and mother warmth were the most consistent predictors of both for girls’ and boys’ LSE and MSE in the Turkish context. As expected, mother warmth had positive and stronger effects on predicting girls’ LSE and MSE, and boys’ LSE. Father warmth had positive and stronger effects on boys’ LSE, and girls’ and boys’ MSE.

### Testing the Role of Co-parenting Quality

We specifically tested the effects of co-parenting quality by creating all possible combinations of co-parenting (in)consistencies. For this purpose, we first created four categories of co-parenting: (a) *positive co-parenting* describes when both mothers and fathers were simultaneously above the mean scores of positive parenting behaviors (i.e., warmth) and below the mean scores of negative parenting behaviors (i.e., rejection, intrusion, and guilt induction). On the other hand, (b) *negative co-parenting* describes when both mothers and fathers were simultaneously above the mean scores of negative parenting behaviors and below the mean scores of positive parenting behaviors. Finally, (c) *inconsistent co-parenting* (it could also be called asymmetric or lack of co-parenting) describes when one of the parents fell above the mean scores while the other one was below. Two types of inconsistent coparenting were created. One refers to the condition in which the given maternal behaviors were above and the paternal behavior was below the mean scores, and the other refers to the opposite pattern. Thus, children were divided into four groups using mean splits for the given perceived paternal and maternal parenting behavior.

Descriptive analyses showed that the majority of children had positive co-parenting ranging from 725 to 1,083 across parenting behaviors. The number of children under negative co-parenting conditions was relatively low, ranging from 383 to 649. The number of children in group 3 (mother above, father below the mean) under inconsistent co-parenting conditions ranged from 195 to 307. Last, the number of children in group 4 (mother below, father above the mean) under inconsistent co-parenting conditions ranged from 221 to 241. The number of children under positive co-parenting conditions was always highest in all parenting behaviors. This was followed by negative co-parenting conditions, similarly for all parenting behaviors. With minor differences, the number of children in group 3 was higher in warmth, intrusion, and guilt induction behaviors compared to that in group 4.

To interpret the findings based on the classification given above, we specifically defined the compensation and deterioration effects for positive and negative parenting behaviors as follows: the compensation effect for warmth was observed when inconsistent parenting (i.e., one of the parents had a higher and the other had a lower level of warmth) was not significantly different from positive co-parenting (i.e., both parents have higher levels of warmth). Conversely, the deterioration effect for warmth was observed when inconsistent parenting yielded significantly lower levels of academic self-efficacy than positive co-parenting. This pattern is reversed for negative parenting behaviors (i.e., rejection, intrusion, and guilt induction). Specifically, the compensation effect was observed when children’s level of academic self-efficacy in the inconsistent co-parenting groups was not significantly different from that in the positive co-parenting groups (i.e., both parents have lower levels of negative behaviors). Lastly, the deterioration effect for negative parenting behaviors was observed when the levels of outcome variables in the inconsistent co-parenting groups were significantly lower than those in the positive co-parenting groups. Thus, we set the positive co-parenting group as the reference group in determining the compensation and deterioration effects.

We performed univariate ANOVAs on the groups (1, 2, 3, and 4) on LSE and MSE separately for girls and boys on all parenting behaviors. We conducted a *post hoc* analysis with Tukey test if the effect was significant. As presented in Table 3, for the effect of warmth on LSE, results revealed a significant main effect of co-parenting quality on the girls’ and boys’ LSE. Children in group 1 (positive co-parenting) had the highest and those in group 2 (negative coparenting) had the lowest levels of LSE. Girls in group 3 (inconsistent co-parenting [mother above, father below the mean]) had higher levels of LSE, which was similar to positive co-parenting and different from negative co-parenting, indicating a mother compensatory effect for girls. Boys in group 3 (inconsistent co-parenting [mother above, father below the mean]), however, had a lower level of LSE than those in group1 (positive co-parenting), indicating a father deterioration effect.

**TABLE 3:** Literature and math self-efficacy scores of girls and boys across the four groups of co-parenting (in)consistency.

	Literature self-efficacy							
	
	Girls (*N* = 957)							

Sub-groups	1	2	3	4	*MS*	*F*	$\eta_{\text{p}}^{2}$	Effect type
Warmth	3.51_a_	3.21_b_	3.40_ac_	3.34_bc_	5.34	23.75**	0.07	Mother compensation
Rejection	3.24_a_	3.46_b_	3.30_ac_	3.40_bc_	2.44	10.43**	0.03	Mother compensation
Intrusion	3.30_a_	3.44_b_	3.38_ab_	3.40_ab_	0.89	3.73*	0.01	No effect
Guilt induction	3.42	3.41	3.34	3.34	0.35	1.44	0	No effect
	**Boys (*N* = 933)**							
Warmth	3.38_a_	2.99_b_	3.10_bc_	3.18_c_	9.3	37.92**	0.11	Father deterioration
Rejection	3.03_a_	3.30_b_	3.09_a_	3.15_a_	4.29	16.41**	0.05	Inconsistency deterioration
Intrusion	3.14	3.24	3.19	3.15	0.66	2.43	0.01	No effect
Guilt induction	3.27_a_	3.16_b_	3.14_ab_	3.19_ab_	0.89	3.28*	0.01	No effect

	**Math self-efficacy**							

	**Girls (*N* = 957)**							

Warmth	3.23_a_	2.89_b_	3.02_b_	3.05_b_	6.89	22.86**	0.07	Inconsistency deterioration
Rejection	2.88_a_	3.19_b_	2.99_a_	3.02_a_	5.05	16.42**	0.05	Inconsistency deterioration
Intrusion	2.95_a_	3.18_b_	3.01_a_	3.06_ab_	2.96	9.42**	0.03	Mother deterioration
Guilt induction	3.13	3.09	3.02	3.1	0.49	1.52	0.01	No effect

	**Boys (*N* = 933)**							

Warmth	3.30_a_	3.05_b_	3.13_b_	3.19_ab_	3.78	12.91**	0.04	Father deterioration
Rejection	2.97_a_	3.28_b_	3.22_b_	3.16_b_	4.77	16.48**	0.05	Inconsistency compensation
Intrusion	3.08_a_	3.27_b_	3.12_a_	3.16_ab_	2.13	7.16**	0.02	Mother deterioration
Guilt Induction	3.22	3.19	3.13	3.18	0.31	1.03	0	No effect

*p < 0.01, **p < 0.001.

*Children were categorized into four groups using mean splits on the basis of the given maternal and paternal parenting behavior. Group 1 represents both mother and father ratings are above their group means; group 2 represents both mother and father ratings are below the group means; group 3 represents mother rating is above and father rating is below the group mean; and group 4 represents mother rating is below the group mean and father rating is above the group mean. Post hoc differences among the groups were calculated using Tukey’s honestly significant difference (HSD). Means not sharing subscripts differ significantly at the level of α = 0.05, as indicated by Tukey’s HSD (see section “Testing the Role of Co-parenting Quality” for detailed descriptions of the subgroups).*

For the effect of warmth on MSE, there were significant group differences both for girls and boys. Girls and boys in group 1 (positive co-parenting) reported the highest, and in group 2 (negative co-parenting) reported the lowest levels of MSE. Girls in group 3 and group 4 (inconsistent co-parenting) reported a lower level of warmth than those in the positive co-parenting group, suggesting an inconsistency deterioration effect for both mothers and fathers. Boys in group 3 (inconsistent co-parenting [mother above, father below the mean]) reported a lower level of warmth than those in the positive co-parenting group, suggesting a father deterioration effect.

For the effect of rejection on LSE, results again revealed a significant main effect of co-parenting quality. The level of LSE was highest in group 2 (positive co-parenting) and lowest in group 1 (negative co-parenting) among both boys and girls. There was no significant difference between group 4 (inconsistent co-parenting [mother below, father above the mean]) and group 2 on girls’ LSE, suggesting a mother compensation effect. However, boys in negative and inconsistent co-parenting groups reported lower levels of LSE than those in positive parenting group, suggesting an inconsistency deterioration effect for both mothers and fathers.

For the effect of rejection on MSE, girls and boys in group 2 (positive co-parenting) reported highest levels of MSE. There was no significant difference between the negative and inconsistent coparenting groups on girls’ MSE. However, there was a significant difference between the inconsistent and positive co-parenting groups in boys’ MSE. These results suggested an inconsistency deterioration effect for the girls and an inconsistency compensatory effect for the boys.

Regarding the effect of intrusion on LSE, there was no significant group differences for boys. Girls in group 2 (positive co-parenting), however, had the highest, whereas girls in group 1 (negative co-parenting) had the lowest levels of LSE. No compensation or deterioration effect was observed.

For the effect of intrusion on MSE, results were significant for both girls and boys. Again, girls and boys in group 2 (positive co-parenting) had highest levels of MSE. *Post hoc* results showed that girls and boys in group 3 (inconsistent co-parenting [mother above, father below the mean]) reported a lower level of intrusion than those in group 2 (positive co-parenting), suggesting a mother deterioration effect for both girls and boys.

For the effect of guilt induction, results revealed significant differences between co-parenting groups only for boys’ LSE levels. No effect was found for girls’ LSE and girls’ and boys’ MSE. Boys in group 1 (negative co-parenting) had higher levels of LSE than those in group 2 (positive co-parenting). These results suggested that guilt induction (although it was conceptually negative parenting) had a positive effect on boys’ LSE. No compensation or deterioration effect was observed.

Overall, as expected, children with positive co-parenting had the highest, and those with negative co-parenting had the lowest levels of academic self-efficacy with one exception. Contrary to our expectation, boys reporting higher levels of guilt induction also had a high level of LSE. Inconsistent co-parenting yielded compensatory or deterioration effects. Moreover, parental warmth had moderately strong effect sizes, with $\eta_{\text{p}}^{2}$ values ranging from 0.04 to 0.11. As subdimensions of culture-common parenting behaviors, intrusion and guilt induction yielded weak effect sizes, with $\eta_{\text{p}}^{2}$ values ranging from 0.01 to 0.03.

## Discussion

This study investigated the unique contribution of fathers’ perceived parenting behaviors over mothers’ behaviors and the quality of co-parenting in primary school children’s academic self-efficacy. As expected, both girls’ and boys’ literature and math self-efficacy increased as a function of high levels of positive parenting behaviors and low levels of negative parenting behaviors. Importantly, the number of father and mother variables that remained significant in the regression models were close. This shows that fathers’ parenting was as effective as mothers’ parenting. In addition, a series of ANOVAs testing the effects of coparenting consistency showed that children with positively consistent parents reported the highest levels of academic self-efficacy, and that those with negatively consistent parents reported the lowest level of academic self-efficacy. Combinations of inconsistent co-parenting, however, revealed mother and father compensatory and deterioration effects depending on the parent’s and child’s gender, domain of parenting behavior, and academic efficacy. Overall, parental warmth was the strongest predictor in regression analysis. As we expected, the effects were weak or non-significant for the culture-common psychological control variables, intrusion, and guilt induction. These observations are discussed as a function of child’s and parent’s gender, parenting behaviors, and outcome variables.

We specifically focused on children’s perceptions of universal and culture-common parenting behaviors in predicting academic self-efficacy. As seen in Table 1, on a four-point scale, mean scores of the parental emotional warmth are highest, whereas parental rejection is lowest for both fathers and mothers. This shows that parents in Turkish culture are likely to adopt functional levels of universal parenting behaviors. Regarding culture-common parenting practices reflecting the specific dimensions of parental psychological control, the mean of perceived guilt induction was relatively higher than the mean of intrusion. This suggests that Turkish parents may see guilt induction as a way of securing emotional interdependence or constant relatedness of their children (Kağıtc̨ıbas̨ı, 2007; Sümer and Kağıtc̨ıbas̨ı, 2010). Consistently, children may perceive their parents’ guilt induction behavior as an indicator of parental emotional warmth and involvement in the given cultural context. Interestingly, children did not perceive high levels of intrusion from their parents. This might again imply that children perceived their parents’ use of intrusion as normative given the collectivistic values of Turkish culture. Alternatively, the parents in this sample did not adopt high levels of psychological control but showed a trend for more adaptive parenting strategies.

Another important finding on the mean level analyses was gender difference between girls and boys in academic self-efficacy and perceived parenting behaviors. In line with the literature, the girls had higher literature self-efficacy, and the boys had higher math self-efficacy (Marsh and Craven, 2006). The difference between the girls and the boys was stronger in literature self-efficacy, which demonstrates that the girls are more confident in their literacy skills. In addition, the boys seemingly reported higher levels of perceived negative parenting behaviors (i.e., rejection, intrusion, and guilt induction), which can be interpreted as boys’ greater demand for autonomy than girls, yet the girls tended to report higher levels of warmth than the boys.

We tested our expectations on the unique contribution of fathers’ parenting behaviors over and above mothers’ parenting behaviors by examining the number of significant effects that remained in the models and comparing the *standardized beta* values. Results revealed that four significant mother effects and three significant father effects remained significant in the second step of hierarchical regression models. Although the numbers were similar, it does not equate to the roles of fathers and mothers. To begin with, we found a consistent positive effect of parental warmth on children’s academic self-efficacy. Although mother warmth revealed a clear and strong effect on literature self-efficacy both for girls and boys, it was different in math self-efficacy. That is, there was only a significant effect of mother warmth on girls’ math self-efficacy. Father warmth, however, yielded significant effects on math self-efficacy both for boys and girls. Moreover, father warmth significantly or marginally significantly remained in the other models even after controlling for the mother effects. These positive effects showed that both mother warmth and father warmth are critical and needed for positive child outcomes (Pinquart, 2016). However, mother warmth seemed to be more important for girls and literature self-efficacy, and father warmth seemed to be more important for boys and math self-efficacy in the Turkish cultural context.

Regarding parental rejection, we expected that this universally negative parenting dimension would decrease children’s academic self-efficacy. Maternal rejection seemed to deteriorate child outcomes, especially for girls. However, these effects were not significant in the second step of the models, except that maternal rejection had a marginally significant effect on girls’ math self-efficacy. Overall, comparison of the father and mother effects in the universal parenting dimensions demonstrated that the mothers had a greater number of significant effects, but that fathers’ effects were relatively larger in the size of beta values, although these betas were not statistically compared. Our findings were in line with past studies showing the importance of fathers as well as mothers (Kim and Rohner, 2002). We can argue that fathers and mothers might function differently (Chen et al., 2000; Lv et al., 2018) and make their contributions in their unique ways (Jeynes, 2016).

The power of culture-common parenting behaviors in predicting children’s academic self-efficacy was weak. Maternal intrusion only negatively predicted girls’ MSE. There were no other significant effects of intrusion and guilt induction. This suggests that similar to the findings in other collectivistic cultures, such as that in China (e.g., Chen et al., 1998), these aspects of psychological control might be perceived as normative; hence, it has fewer negative effects on child functioning in Turkish culture. Consistent with previous findings showing significant relationships between general parenting practices and styles and children’s academic concept or achievement (Suizzo et al., 2017), we found that the universally positive parenting behavior, namely warmth, had the strongest effect. It should be noted that parenting behaviors specific to academic domains such as parents’ educational involvement (Catsambis, 2001) and academic aspirations (Lv et al., 2018) explain more variance in academic efficacy than general parenting behaviors. Besides, this lack of significant findings draws attention to cultural interplays of psychological control. That is, culturally common and relevant parental psychological control behaviors were not perceived as negative in Turkish culture. Consistently, a previous study has shown that parental psychological control and attachment insecurity are not associated in the Turkish cultural context (Güngör and Bornstein, 2010; Sümer and Kağıtc̨ıbas̨ı, 2010). This study expanded this effect to the domain of academic self-efficacy.

Beyond the unique role of fathers in child development, how well fathers cooperate with mothers is a critical factor. This study extended the definition of co-parenting to the consistency between parenting behaviors. In line with this, three types of co-parenting were specified, namely, positive coparenting, negative coparenting, and inconsistent coparenting. Our expectations on positive co-parenting overlap with those of a previous study (e.g., Teubert and Pinquart, 2010). Positive co-parenting represents the optimal level of agreement, consistency, and similarity in child-rearing strategies; hence, it is the most functional co-parenting type among all. Our study has shown that children who are raised in a positive co-parenting climate have the highest level of literature and math self-efficacy. Conversely, negative co-parenting behaviors led to the lowest levels of academic self-efficacy, implying that above the unique effects of maternal and paternal parenting, the quality of co-parenting seems to have an additional advantage, which should be inquired about more in further studies.

The only exception that was inconsistent with the effects of positive and negative co-parenting types was the effect of guilt induction on boys’ literature self-efficacy. Specifically, boys who perceived higher levels of guilt induction from both parents had the highest level of literature self-efficacy, although effect size was minimal. We can speculate that children’s perceptions of parenting behaviors are much more important than actual parenting. When children interpret high levels of parenting psychological control as an indication of parental love and care, the negative effects of these behaviors may lessen (Scharf and Goldner, 2018). This is not conclusive for this study, since we did not measure children’s perceptions of normativeness of these parental behaviors. Furthermore, the adverse effects of parental controlling behaviors may decrease as a function of socialization goals in collectivistic cultures. Parents may benefit from the means of psychological control, such as guilt induction, as a teaching strategy, imposing cultural values, or raising empathy in their children toward themselves and others (Scharf and Goldner, 2018). Therefore, perceptions of higher levels of guilt induction from both parents may create an opportunity for increased levels of self-efficacy. Our findings are in line with cultural interpretations of parenting behaviors; however, more research is needed.

Although we did not statistically perform any interaction analysis, we can speculate on joint effects of variables looking at the number of significant effects. In line with previous studies (e.g., Ryan et al., 2006; Baril et al., 2007; Feinberg et al., 2007; Cabrera et al., 2012), inconsistent co-parenting revealed compensation or deterioration effects depending on parent and child gender, domain of parenting behavior, and academic efficacy. Inconsistent co-parenting was compensatory for children only to some degree. As seen in Table 3, out of 10 significant effects of inconsistent co-parenting, three are compensatory effects. However, one can argue that some of these compensation effects can also be seen as deterioration depending on interpretation. For instance, for the effect of warmth on girls’ LSE, we observed a mother compensation effect, since reporting higher levels of mother warmth yielded higher levels of LSE, which indicated a mother compensation effect. On the contrary, reporting lower levels of mother warmth yielded lower levels of LSE, which indicates a mother deterioration effect, which indeed confirms the mother’s critical role. A similar interpretation can also be made for the effect of rejection on girls’ LSE. We call for careful interpretation of this situation but still suggest that having one parent may be good enough to protect a child’s academic self-efficacy from potential detriments of the other careless parent. Consistent with the previous findings, having at least a supportive mother or father benefits children’s cognitive development over having negative coparenting (Ryan et al., 2006).

The inconsistency compensatory effects showed a high level of match between the gender of parents and that of the children. There were two significant mother compensatory effects, and these were for the girls. There was one significant inconsistency compensatory effect, and this one was for the boys. This suggests that having one parent with optimal level of parenting behavior was enough for boys to create a compensatory effect regardless of the gender of the parent. These numbers point to a tendency for same-sex parent–child compensatory effects, particularly for girls’ academic self-efficacy. Previous studies have provided mixed findings on this issue. For instance, McGrath and Repetti (2000) found that when mothers were satisfied with their children’s performance, both daughters and sons reported high levels of academic self-perceptions. However, when fathers were similarly satisfied with their children’s academic performance, only boys reported high self-perceptions. Again, mother warmth was strongly associated with girls’ academic achievement, but both mother warmth and father warmth were related to boys’ achievement (Pinquart, 2016). The amount of time spent between mother-daughter and father–son dyads is generally higher than the amount of time spent in mixed-sex parent–child dyads (Maccoby, 2003), suggesting a stronger socialization effect for the same-sex parent–child dyads in the academic domain.

There were seven deterioration effects in total, and three of these were observed for the girls and four were observed for the boys. One important finding was that deterioration effects were more prevalent in the parental emotional warmth and rejection domains. Regarding the effect of literature self-efficacy, the boys do not seem to tolerate the effect of having one parent showing higher levels of rejection although the other parent was not rejecting. The same inconsistent deterioration effect was observed for girls’ math self-efficacy. In sum, negative co-parenting influenced boys’ and girls’ academic self-efficacy similarly. Inconsistent co-parenting, especially inconsistency in perceived emotional warmth and rejection, seems to predominantly deteriorate girls’ math self-efficacy.

Overall, the girls seemed to be more open to the effects of perceived parenting and co-parenting behaviors, particularly in math self-efficacy. The general belief about girls’ and boys’ academic competence is that girls are more successful in language and related areas, and that boys are more successful in math and related areas (Pajares, 2002). Parents or teachers might share this biased assumption (Eccles et al., 1990; Voyer and Voyer, 2014). These beliefs, as a result, might create a gender difference in children’s perceptions about their skills (Marsh, 1993; Parker et al., 2018). This study provided convergent results. As stated, the girls were higher on literature and the boys were higher on math self-efficacy. That said, a greater number of parenting behaviors (*N* = 3) predicted girls’ math self-efficacy compared to their literature self-efficacy (*N* = 1), and the number of deterioration effects was higher in girls’ math self-efficacy (*N* = 3) than in any other group. These findings, together, imply that girls represented a more sensitive profile of academic self-efficacy, and that this sensitivity was highly apparent in their math self-efficacy.

Although this study improves our understanding of the role of fathers, we should note several limitations. First, we used only child perceptions to measure the effect of parenting behaviors. Future research should also employ parents’ reports of parenting behaviors and practices. Second, we examined the effect of parenting behaviors in four domains only. Future studies should test the effect of parenting and co-parenting with other dimensions, such as autonomy granting. Third, we had relatively low reliability values of parenting measures, particularly for culture-common parenting behaviors. We had fewer items to measure culture-common practices (i.e., guilt induction) that represent diverse guilt-inducing practices of Turkish parents. This might be one of the reasons for the inconsistent effects of guilt induction, especially on boys’ LSE levels. Future studies should attempt to replicate these findings with more robust and culturally relevant measures of psychological control dimensions. Besides, deterioration and compensation effects should be interpreted with caution, since the inconsistent co-parenting groups did not statistically differ from each other. Finally, we used a very large sample size and four-point Likert scales, which might have decreased the size of correlations, although they remained statistically significant.

This study contributes to the extant research on fathering by assessing the unique role of fathers and co-parenting behaviors in primary school children’s literature and math self-efficacy. Previous studies have mostly focused only on one parent who is generally the mother. However, the understanding about parenting should move forward in new directions; thus, fathers are needed to be more involved and visible in child development. The findings of this study suggest that the effect of one parent is not superior to the other considering that the number of significant effects for mothers and fathers was similar although the magnitude of the effects slightly varies. Still, it does not underestimate the unique importance of fathers or mothers. As seen in the clear superiority of positive co-parenting effects, the presence and harmony of both parents create an optimal climate for high academic self-efficacy. This is particularly valuable for same-sex parent–child dyads. This study also marks that parental emotional warmth, as the universally positive parenting behavior, together with positive coparenting had the strongest positive effect on academic self-efficacy.

Our findings also have several practical implications, particularly in the development of parenting programs and policies. We know that academic self-efficacy is the motivational source of school success and contributes to children’s academic achievement in the long run (Marsh and Martin, 2011). Thus, parenting intervention programs should especially focus on parental consistency and cooperation in specific domains of parenting behaviors and practices, since these are strongly related to academic self-efficacy. This study provides evidence that practitioners, teachers, or educational policymakers can focus on positive co-parenting practices to promote gains in academic efficacy and achievement as a general and fundamental strategy.

## Data Availability Statement

The raw data supporting the conclusions of this article will be made available by the authors, without undue reservation.

## Ethics Statement

The studies involving human participants were reviewed and approved by Middle East Technical University, Human Research Ethics Committee. Written informed consent to participate in this study was provided by the participants’ legal guardian/next of kin.

## Author Contributions

NS was the principal investigator of the project, provided the data for the current study, double-checked the analysis, and reviewed and edited the whole manuscript. NS and DK contributed to the conceptualization and design of the study. DK organized the database, performed the statistical analysis, and wrote the first draft of the manuscript. Both authors contributed to the manuscript equally and approved the submitted version.

## Conflict of Interest

The authors declare that the research was conducted in the absence of any commercial or financial relationships that could be construed as a potential conflict of interest.

## Publisher’s Note

All claims expressed in this article are solely those of the authors and do not necessarily represent those of their affiliated organizations, or those of the publisher, the editors and the reviewers. Any product that may be evaluated in this article, or claim that may be made by its manufacturer, is not guaranteed or endorsed by the publisher.
